# Willingness to Pay to Improve Quality of Public Healthcare Services in Mauritius

**DOI:** 10.3390/healthcare10010043

**Published:** 2021-12-27

**Authors:** Jamiil Jeetoo, Vishal Chandr Jaunky

**Affiliations:** 1 Open University of Mauritius, Reduit 80837, Mauritius; jamiil.jeetoo@gmail.com; 2 Department of Business Administration, Technology and Social Sciences, Luleå University of Technology, SE-971 87 Lulea, Sweden

**Keywords:** contingent valuation, double-bounded dichotomous choice, healthcare services

## Abstract

Mauritius has a universal free healthcare system, based on the Beveridge model which is financed by taxpayers. There are growing considerations over improving quality of healthcare services. The purpose of the study is to employ a contingency valuation (CV) to investigate the willingness of Mauritians people to pay to improve the quality of public healthcare services and the associated determinants using the double-bounded dichotomous choice model. A drop off survey with a sample size of 974 respondents from the working population is used. The empirical analysis shows that the majority of the sample was willing to pay for improving quality of public healthcare services. Other than the conventional determinants of respondents’ demographic and socioeconomic characteristics, the findings support the assertion that psycho-social constructs such as the Theory of Planned Behaviour, Norm-Activation, Public Good Theory, and Perceived Response Efficacy are found to significantly affect Willingness-to-Pay (WTP). The results of this study might be of use to policymakers to help with both priority setting and fund allocation.

## 1. Introduction

The national healthcare system in Mauritius operates on a dual-track basis encompassing the public and the private sectors. Mauritius has one among the most expensive healthcare systems in Africa. Around 73% of the healthcare needs of the population are managed, free of any user cost, at the point of use, in the public sector, financed by the Beveridge system [1]. Under this model, the government raises revenue through taxes and other means, to finance the delivery of social services, including health. The remaining 27% of healthcare needs are dealt with in the private sector, on a fee basis, either through out-of-pocket payments, including deductibles or payments effected by private health insurers. It is highlighted that patients who see the public sector providers could also use the private sector service, and vice versa.

From the National Health Accounts Report 2017 of the Ministry of Health and Quality of Life [1], in the public healthcare sector, in 2016, the primary healthcare network comprised 18 area health centres, 116 community health centres, 5 medi-clinics and 2 community hospitals. In 2016, 4,732,358 attendances were recorded at the primary healthcare institutions. In the private sector, there were 17 hospitals in 2016, which catered for some 233,966 patients. The private health sector also comprised 30 private medical laboratories, 3 imaging and diagnostic centres and around 342 pharmaceutical retail outlets in 2016. Mauritius spent an estimated total amount of US$559 (Rs 20,023) per capita on healthcare in 2016; out of which general government healthcare expenditure was 43.87% (US$245/Rs 8784 per capita) [1].

**Figure 1:**
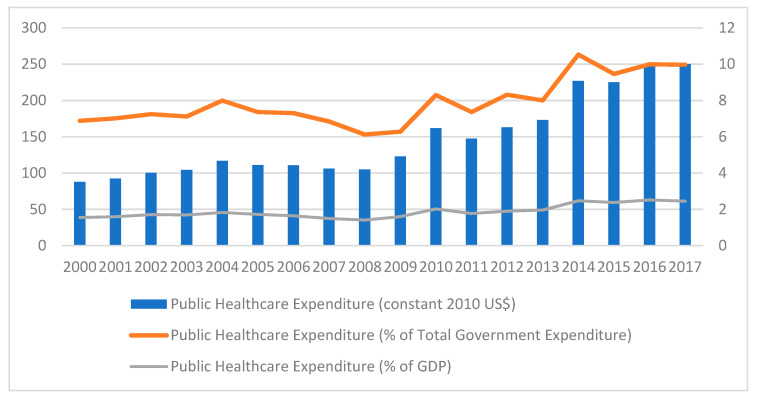
Trend of Public Healthcare Expenditure. Source: Authors’ own computation.

The World Health Report 2000 ranked Mauritius health system performance 84th with a score of 0.691 [2]. According to the 2017 World Health Organization (WHO) Global Monitoring Report, the Universal Health Coverage (UHC) Index for Mauritius was 64 in 2015. Over the last 30 years, life expectancy at birth has increased from 65 years to 74.4 years in 2019 [3]. Further, Mauritius has already achieved some of the health-related Sustainable Development Doals (SDG) targets set for 2030 as given in the table below (Table 1):

Recent years have witnessed an effort to improve health care services, with a significant increase in the allocated budget, ranging from 6.9% of the government’s total budget in 2000 to 10% in 2017 [5]. Figure 1 shows the increasing trend in Public Healthcare Expenditure in real terms (constant 2010 US$) and as a percentage of Total Government Expenditure. Despite the substantial resources that the government is currently able to allocate, the health care system is increasingly under strain as a result of the most pertinent challenges faced by all publicly funded health care systems; rapid increases in expenditures and demand while resources remain finite. These challenges include an ageing population, an increase in sedentary lifestyles, rising costs, increasing user expectations and changing disease patterns [6].

**Figure 2:**
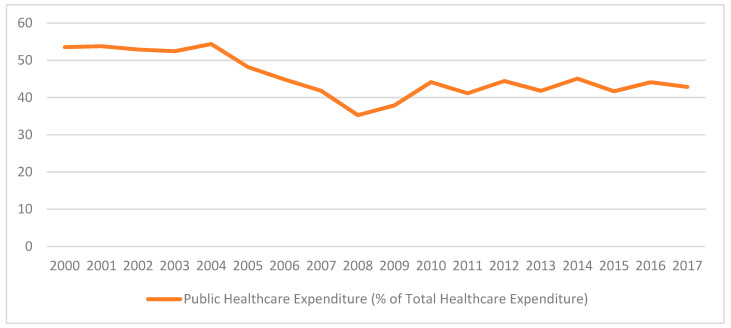
Evolution of Public Healthcare Expenditure as a Percentage of Total Healthcare Expenditure. Source: Authors’ own computation.

Figure 2 shows the trend in Public Healthcare Expenditure as a percentage of Total Healthcare Expenditure. It is noticed that the government has been allocating more resources to the provision of healthcare services in Mauritius. However, it noted that as from 2005, the share of private healthcare financing has outpaced that of public healthcare financing. This tends to indicate that while the Mauritian government has been investing massively in the healthcare sector, many Mauritians opt for private healthcare despite the fact that these are relatively more expensive [7]. As per [8], the public healthcare system is perceived negatively by the general population and some people prefer private healthcare services due to poor quality of services in the public healthcare sector. Consequently, people at the bottom of the socioeconomic ladder obviously cannot access paid services [8].

As per the Ministry of Health and Wellness [3] the Health Sector Strategic Plan (HSSP) 2020–2024 the mission of healthcare institutions is to provide safe and quality healthcare services to patients and to meet their needs and expectations. The government recognizes that the increasing expectations of health consumers for improved quality of care [1]. To this end, expectations of patients are considered to form part of the basic determinants of quality of care (HSSP, 2020–2024).

More recently, improving the quality of services provided to the population is one among the main objectives of the HSSP 2020–2024 [3]. The HSSP 2020–2024 “unveils concrete strategies and interventions to address peoples’ expectations for an enhanced quality of services across their lifespan”. As per the report, “over the years, patients’ expectations for improved quality of healthcare services continue to increase”. Actions proposed in the Health Sector Strategy 2017–2021 lay the basis to further enhance the quality of health care services in response to patients’ growing expectations.

As per the Minister of Health and Wellness [3], the mission of public healthcare institutions is to provide safe and quality healthcare services to patients and to meet their needs and expectations. It is noted that people want a value for money service given that free healthcare services in the public sector are funded through taxation [3]. There is a specific strategic goal (Goal 19) of the HSSP oriented towards “Institutionalizing Health Research to improve quality of healthcare services” in Mauritius. Greater understanding regarding the value and importance of improvements in the healthcare sector is vital for policymakers and this can provide support for increasing healthcare for increasing the fiscal space of the public healthcare financing. The HSSP 2017–2024 highlights the need to invest in improving quality of public healthcare services in Mauritius to keep pace with the “requirements and expectations” of the population. As such, it is useful to explore the value placed on improving the quality of public healthcare services in Mauritius.

Using a CV method, this study aimed to assess the value and importance of improvements in the quality of public healthcare services in Mauritius. CV is a stated preference method that has been widely used to assess public preferences through eliciting the WTP values. It is a hypothetical approach that uses surveys to place economic values on public goods by obtaining information on individual preferences and determining what they would be willing to pay for public goods and services when prices are not available [9,10]. CV can be used to elicit WTP for different purposes, including informing the budget-allocation decisions of publicly financed health care systems, assessing demand, measuring the value of certain aspects or attributes of health care services, and determining the prices of goods to be traded on the market. It can also be used to inform policymakers of the extent and source of other resources that could be mobilized to finance the health care system or health programmes [11,12,13].

The previous studies have focused on the characteristic of demographic and socioeconomic factors such as age, income, and family size. This study contributes by turning to psycho-social constructs to explore theories such as Public Good Theory [14], Norm Activation Model [15] and Theory of Planned Behaviour [16]. The remaining of the paper is organised as follows: Section 2 reviews the literature. Section 3 presents the methodology used for the study. Subsequently, the results are discussed in Section 4. Section 5 concludes with a reflection on the results.

## 2. Literature Review and Conceptual Framework

### 2.1. Theoretical Framework Contingency Valuation and Willingness-to-Pay

In the literature, stated preference methods have been often used to assess public preferences. The most commonly used stated preference approach is Contingency Valuation Method (CVM) which elicits market value of non-market goods. The approach uses survey methods to ask people about their willingness-to-pay (WTP) to obtain a hypothetical good/service (hypothetical scenario) and to place an economic value on same. It aims at collecting information on respondents’ preferences to determine the amount they would be willing to pay for the hypothetical scenario in absence of a market price [9].

CVM can be applied in different contexts to elicit WTP. Adapted to the health economics literature, it can be used to inform decisions about budget-allocation of government funds to health projects, to assess demand for health products or services and to measure the value of certain aspects of healthcare services, including their improvement. It may be used as a tool to advise policymakers of additional sources and potential size of other resources that may be mobilised to finance healthcare projects/programmes [12,13].

The theoretical construct of the CVM is rooted in the theory consumer behaviour. For a given hypothetical scenario, an indirect utility function can be used to derive the WTP. Suppose that a rational individual aims to derive maximum utility from a hypothetical good given the given the quantity, *Q*, and income, *Y*. The corresponding utility function is given as:

*U*(*Z*, *Q*) (1)

where *Z* is a vector of the good.

The problem of an individual is to maximize the utility function *U*(·) subject to the budget constraint,

*PZ* = *Y* (2)

where *P* is a vector of price. Given the level of *Q*, the solution is a combination of Marshallian demand functions,

*Z_i_*(*P*, *Y*, *Q*) (3)

Using the above theoretical concept, the CVM uses indirect utility functions,

(4) $$V_{i}\left(P,M;Q\right):\;\forall_{i}$$

where *i* = 1, 2, …, *I*, representing individuals.

Following the CVM, when the “hypothetical scenario” is implemented, *Q* improves from $Q_{0}$ to $Q_{1}$. The compensating surplus an individual is willing to pay for the improvement, i.e., remains at the same compensated utility level, can be measured as:

(5) $$U_{i}\left(P,Y;Q_{0}\right)=U_{i}\left(P,Q_{1},Y-\mathrm{WTP}\right)$$

The individual’s WTP (${\mathrm{WTP}}_{i}$) for the improvement may be expressed as:

(6) $${\mathrm{WTP}}_{i}=\left(P,Q_{1},\;U_{0}\right)-x\left(P,Q_{0},U_{0}\right)=P\{H\left(P,Q_{1},\;U_{0}\right)-H\left(P,Q_{0},U_{0}\right)\}$$

From the above, *x*(·) denotes the expenditure function and *H*(·) denotes the Hicksian compensated demand function. WTP for health improvement relies on the assumption of maximizing utility and the axioms regarding rational consumer preferences. In line with [17], an individual would be willing to pay a specific amount of money so as to acquire any gain in utility.

### 2.2. Empirical Literature

From the empirical literature, three studies are found to explore the determinants of WTP to improve quality of healthcare using CVM [18,19,20]. However, the studies differ in terms of their sampling data, country of study, methodology used and most importantly factors controlled for in their models. A summary of these studies is provided in Table 2.

Pavel et al. [18] investigate the determinants of WTP for improving quality of seven attributes of healthcare in Bangladesh–more specifically in three hospitals. It uses a sample size of 252 patients and applied the “Partial Tobit Regression” with the corresponding marginal effects. The study has focused on the impact of socio-economic factors on WTP.

Habibov et al. [19] explore the impact of “Social Trust” WTP to improve quality of public healthcare in selected “post-communist countries”. It uses a sample size of 29,526 individuals in 29 post-communist countries and applies the “Classic Binomial Probit and Instrumental Variables Probit Regressions”. In addition to “Social Trust”, the study has included “Political Trust” and socio-economic indicators in the model.

Al-Hanawi et al. [20] analyse the factors affecting WTP to improve quality of public healthcare services in Saudi Arabia. It uses a sample of 1187 household heads in Jeddah and applies the “Partial Tobit Regression”. The study investigates the effect of respondents’ demographic and socioeconomic characteristics, including age, gender, location, marital status, education level, household monthly income, “ownership of private health insurance”, “travel time to reach the public health care services” and “whether any household member suffers from a chronic disease”, as well as “quality attributes of public health care services” including “availability of appointments”, “waiting time before seeing the doctor”, “waiting time for laboratory tests”, “availability of drugs”, “staff attitudes”, “doctor–patient relationship” and “outcome of treatments”, on WTP to improve quality of public healthcare services. Seven regressions corresponding to the various attributes are estimated.

### 2.3. Conceptual Framework and Hypotheses Development

**Figure 3:**
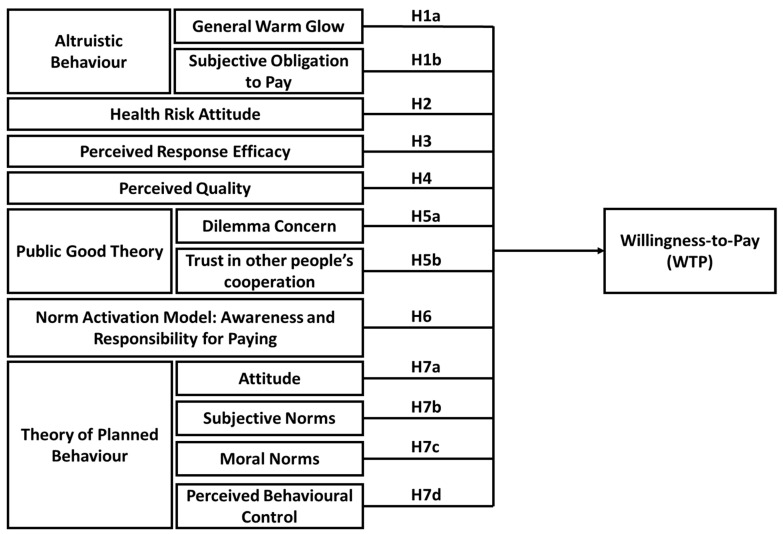
Conceptual Model.

The conceptual model is illustrated in Figure 3. It is derived from the three major theories which we believe are most important in guiding WTP for improving quality of healthcare services, and which have been neglected in previous empirical studies: Public Good Theory [14], Norm Activation Model [15] and Theory of Planned Behaviour [16]. This study defines and develops theoretical support for the presented conceptual model as illustrated in Figure 3.

In line with Jaunky et al. [21], the conceptual framework is closely connected to the questionnaires. This is illustrated in Section 2.12. The hypotheses are explained in the subsequent sections and these are tested from the questions and statements proposed in the questionnaires. The variables used for the hypothesis testing from the conceptual framework are discussed the subsequent section.

### 2.4. Altruistic Behaviour

Altruistic behavioural theories have been included in the framework of contribution models which corresponds to CVM [22,23,24]. Individuals may perceive a moral obligation to support good causes that could benefit the society. Contributions to better healthcare can be one way of gaining personal satisfaction among others. In their WTP study for a public good, Liebe et al. [25] control for altruistic behaviour which they classify as “general warm glow” and “subjective obligation to pay”.

#### 2.4.1. General Warm Glow

In economic models, altruistic motive can be factored in using a utility function which includes “taste for having other people better off” [26]. In this concept, “others” is not necessarily limited to human beings; it can be extended to other “causes”, including the healthcare system [25]. This motive can create a feeling of obligation to pay donate for improving the healthcare system. People can have a general satisfaction such as “to do good,” irrespective of specific part of the healthcare system towards which they are financially contributing to. This concept is based on the fact that people perceive a general obligation to support positive causes and derive utility from same for “whatever reason”. In economic models, this general feel of obligation to pay is often referred to as “a warm glow of giving” [22,27]. It is expected that the “General warm glow” positively affects WTP to improve QPHS.

The first hypothesis proposed is given as:

**Hypothesis** **1a** **(H1a).** *People who perceive a general obligation to support good causes are more willing to pay for improving quality of healthcare services.*

#### 2.4.2. Subjective Obligation to Pay

Another type of altruistic model applicable in economic models is the “Subjective Obligation to Pay”. While under “General Warm Glow” factor, people derive moral satisfaction while from the act of contributing to “a good cause”, “Subjective Obligation to Pay” is based on the fact that this moral satisfaction is depends on the specific project and is not dependent on how better off others will be [26,27]. This is based on the assumption that people derive different utility levels from different projects commonly known as the “embedding effect” [22].

Based on the above, it is expected that both the “Subjective Obligation to Pay” for improving QPHS and the “General Warm Glow” Thus, the following hypothesis is formulated:

**Hypothesis** **1b** **(H1b).** *People who perceive a subjective obligation to pay for health projects are more willing to pay for improving quality of healthcare services.*

### 2.5. Health Risk Attitude

People have different attitudes when faced with a health-related risk. There exists a relation between risk attitude and behaviour [28]. When a person is more adverse towards risks, he tends to accept less risk than someone he is more risk tolerant [29]. As a result, the risk attitude towards health is a vital source of information about medical decision making [29]. It is expected that Health-risk attitude should affect the WTP for improving QPHS. Himmler et al. [30] control the health risk attitude in their study for an early warning system for infectious diseases and find some evidence of a statistically significant impact on WTP. It is expected that health risk attitude should affect the WTP for improving QPHS. The following hypothesis is formulated:

**Hypothesis** **2** **(H2).** *The risk attitude of people affects their WTP for improving quality of healthcare services.*

### 2.6. Perceived Response Efficacy

The concept of perceived response efficacy has been defined as an individual’s belief on the effectiveness of the recommended response [31,32]. It is based on people’s assessment of the efficacy of the recommended solutions [33,34]. This variable has recently been applied in various behavioural studies [35,36]. It is expected that perception that the recommended solution is effective is positively affect WTP, leading to the following hypothesis:

**Hypothesis** **3** **(H3).** *Perceived response efficacy is positively related to the WTP for improving quality of healthcare services.*

### 2.7. Perceived Quality

Based on the theoretical construct of WTP in CVM, the latter is a function of the improvement level; i.e., the difference between levels before and after a policy scenario, amongst other factors [37]. Hynes et al. [38] defines this improvement as the movement from the status quo to an alternative status.

In a CVM assessment, it is important to assess the perception of status quo of each respondent (i.e., the perceived quality of public healthcare services) as the status quo quality level varies for different individuals, resulting in varying levels of quality improvements, based on the status quo QPHS level [39]. As per Whitehead [39] omitting perceived quality may cause bias estimates in a model. As such, it is important to include a proxy measure for quality in modelling WTP, in line with other studies [18,20] which have followed this approach. It is expected that as satisfaction with current QPHS increases WTP. Perceived quality has been used in various studies related to consumer behaviour such as Johnson and Kellaris [40], Ophuis and Van Trijp [41], Nikhashemi et al. [42] and so on. Therefore, the hypothesis below is formulated:

**Hypothesis** **4** **(H4).** *Perceived Quality of Public Healthcare Services is negatively related to the WTP for improving quality of healthcare services.*

### 2.8. Theory of Public Goods

Individuals can perceive QPHS as a public good and therefore the theories of public goods and collective action is applicable [43,44]. Public goods have the characteristic of non-excludability. This implies that no one can be excluded from its usage once it is provided. As such, this provides an incentive for people to free ride on the contribution of others leading to the issue of “social dilemmas”—the free rider hypothesis. This is based on the fact that “individual rationality leads to collective irrationality” where no one end up paying for a public good-“zero contribution thesis” [45].

Nevertheless, there is a weaker version of the free-rider hypothesis where it is postulated that at least some people would be contributing—though the outcome will still be sub-optimal [46]. From the literature, it is shown empirically that people do not use all opportunity to free ride; they do “cooperate in public good games” [47,48]. This deviation from the economic assumption behind public goods is based on the concept of “conditional cooperation”; i.e., people base their behavioural decision on how others behave [49]. Liebe et al. [25] have applied the public goods theory and provide evidence of its significance in assessing WTP. In similar fashion, based on the above, there are two underlying concepts affecting WTP: (i) dilemma concern and (ii) trust in other people’s cooperation.

#### 2.8.1. Dilemma Concern

Dilemma concern is a concept which captures the degree people view a hypothetical scenario as a social dilemma and it is based on the notion of conditional cooperation [50] Higher consideration for a hypothetical scenario to be a social dilemma is likely to increase WTP.

Therefore, the following hypothesis is proposed:

**Hypothesis** **5a** **(H5a).** *The more people improvement in the healthcare service as a social dilemma, the less likely they are willing to pay for its improvement.*

#### 2.8.2. Trust in Other People’s Cooperation

The notion of “trust in other people’s cooperation” relates to the perception that other people are willing to pay “to do their share”. This is based on the notion of “conditional co-operation” where people are more likely to cooperate if others cooperate [49]. When people trust that others are going to pay, they are likely to believe that they are not the only one who are going to pay and they are more likely to pay [51,52,53]. This suggests the following proposed hypothesis:

**Hypothesis** **5b** **(H5b).** *People who trust that others will cooperate are more willing to pay for improving quality of healthcare services.*

### 2.9. Norm-Activation Model

The norm-activation model, developed by Schwartz [15], explains how a personal norm leads to a moral obligation to perform or refrain from a specific action. As per the theory, a person sacrifices his self-interest for the joint benefits of others [54]. The theory has often been used to predict pro-environmental behaviours [55,56]. According to Schultz et al. [57], the link between personal norm and a specific behaviour is affected by an individual’s awareness of negative consequences and how they ascribe the responsibility. In other words, the model is reflected by the “awareness of need” and “awareness of responsibility” [25]. Norm-activation model has been employed in several WTP studies [24,25,58,59]. Based on the above, when people have higher awareness of the need to pay and higher awareness of the responsibility to pay, this has a positive impact on WTP. Therefore, the current study hypothesizes that:

**Hypothesis** **6** **(H6).** *People with higher awareness of the need and responsibility for paying to improve quality of healthcare services are more likely to pay for its improvement.*

### 2.10. Theory of Planned Behaviour

The Theory of Planned Behaviour (TPB) is an extension of the Theory of Reasoned Action (TRA). As per the latter, the intention to perform a behaviour affects the behaviour of an individual. This intention is intern affected by the “attitude towards the behaviour” and “subjective norms” [60]. The extension of the TRA, which is the TPB, adds “perceived behavioural control” to the equation to improve the predictive power of the TRA [60].

**Figure 4:**
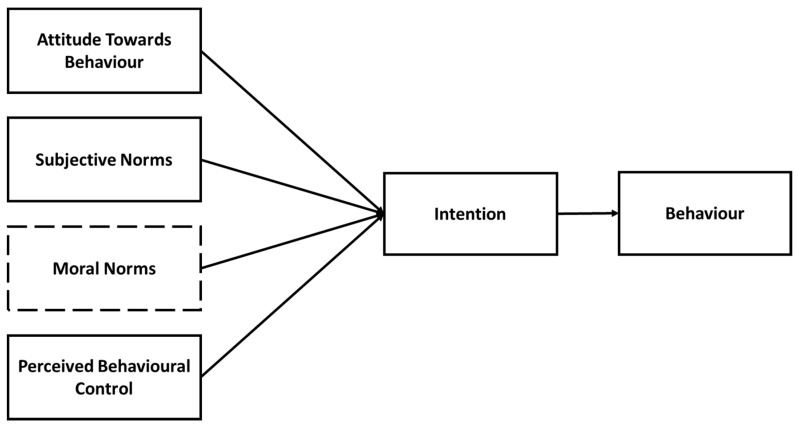
Components of the TPB and their influence on the final behaviour. Source: Adapted from Fishbein and Ajzen [16].

The main notion behind the TPB is that a person’s behavioural intention affects the final behaviour of an individual and is shaped by the person’s attitudes, social norms and perceived behavioural control regarding the behaviour in question. Attitudes refers the whether the final behaviour is viewed a positive or negative. Social Norms mean the perceived social pressure in engaging in the behaviour and Perceived Behavioural Control refers to the easiness or difficulty to perform the behaviour [60]. Ajzen [61] upgraded the theory to include moral norms. The latter signifies the moral satisfaction derived when performing a specific behaviour [62]. Theory of Planned Behaviour has been extensively used in WTP studies [25,62,63,64]. Figure 4 illustrates the TPB as to how Attitudes, Subjective Norms, Moral Norms and Perceived Behavioural Control affects behavioural intentions which in turn affects the final behaviour.

Given the above, this study tests the following hypotheses:

**Hypothesis** **7a** **(H7a).** *As attitudes towards payment for the improvement of quality of public healthcare services become more positive, a person’s WTP increases.*

**Hypothesis** **7b** **(H7b).** *As Subjective Norms regarding payment for the improvement of quality of public healthcare services become more positive, a person’s WTP increases.*

**Hypothesis** **7c** **(H7c).** *As Moral Norms concerning payment for the improvement of quality of public healthcare services become more positive, a person’s WTP increases.*

**Hypothesis** **7d** **(H7d).** *As Perceived Behavioural Control of payment for the improvement of quality of public healthcare services become more positive, a person’s WTP increases.*

### 2.11. Control Variables

It is also important to controls for demographic and socioeconomic characteristics including age, gender, residential area, education level, income level, family size, civil status and ownership of private health insurance [18,20].

### 2.12. Data Gathering

#### 2.12.1. Survey Design and Implementation

A drop-off CVM questionnaire was applied to collect data from working individuals in Mauritius, over the period October 2020–December 2020). The sample includes respondents from both urban and rural areas to ensure representative range of individuals’ perspectives. The drop-off survey method was chosen due to the recommended sanitary measures amidst the COVID-19 situation. In this study, the survey questionnaires were hand-delivery of drop-off surveys to people from the working population [65].

The advantage with the drop-off survey is that it minimizes human interaction compared to the interview method given that the study was conducted during the COVID-19 period in Mauritius. This method is more cost effective than phone interview survey. In additional drop-off surveys are known to have higher response rate than online or email surveys, which also run the risk of self-selection bias [66].

#### 2.12.2. Bias Control

The survey strategy controlled for potential issues regarding selection and response bias, including (i) Interviewer Bias; (ii) Social Desirability Bias; and (iii) Sample Selection Bias.

Firstly, interviewer bias refers to the distortion of response due to the person who questions a participant in research. This may occur when the opinions or expectations of the interviewer influence the response of the participant [67]. Drop-off survey requires self-administered survey forms. Under this method, the respondent is isolated before they start to fill in the questionnaire. This ensures that there is no social clue from the researcher that may affect the respondent. As such, drop-survey technique removes “interviewer bias”. Secondly, social desirability occurs when participants provide an answer which they consider to be more “socially acceptable” or “socially desirable” by others than their “true” answer would have been [66,68]. Given that in drop-off survey, the participants respond to the survey questionnaire in the absence of the researcher/interviewer, this reduces social desirability bias [69]. In this study, a statement of anonymity was clearly provided on the survey questionnaire and it was ensured that only respondents who were unknown and has no direct relationship with the survey collectors were chosen at random for the study sample. In addition, the respondents were made satisfied that there was no way for the researcher to identify the respondent of a particular survey questionnaire. Thirdly, sample selection bias may arise when mostly interested individuals to the subject of concern of the survey questionnaire respond [70]. In this study, sample selection bias was reduced as respondents were chosen at random and the survey questionnaire was given to individuals from all regions and socio-economic groups of Mauritius [71].

#### 2.12.3. Sampling

In its limitation, Al-Hanawi et al. [20] suggests that “a study that seeks to elicit the individual’s or patient’s WTP for quality improvements of public health care services will allow for a better generalisation of the [its] findings”. Pavel et al. [18] already assess WTP for healthcare quality improvements using a sample of patients. This study focuses on individuals who are in the working population who are the income earners in Mauritius.

This study focuses on individuals who are in the working population who are the income earners in Mauritius. In 2019, the working population of Mauritius was estimated to be 551,300, based on the results of the “Continuous Multi-Purpose Household Survey” of Statistics Mauritius [72]. The Raosoft [73] sample size software was used to estimate the sample size required for the study based on the following: acceptable margin of error of ±5%, confidence level of 99% and response distribution of 50%. Based on the working population size of 551,300 individuals, the minimum recommended sample size is 663 participants.

As per Bateman et al. [13], a sample of about 500–1000 is recommended in the event of a closed ended contingent valuation study, which includes the use of dichotomous choice. Further, the use of large sample size avoids bias in the bid design in DBDC models [74,75]. Considering the benefits of large sample size to increase the quality of CV using DBDC models, it was decided to take a sample of 1000 respondents to provide for non-usable questionnaires.

Out of the total, 974 valid survey questionnaires were gathered, while the rest were considered as invalid due to incomplete information or protest responses. Protest response have been excluded to avoid “concept inconsistence” and “underestimation of the WTP value” [9,37,76].

Note that a pilot test was conducted in during the month of August 2020 using 100 questionnaires to estimate the average WTP for improving QPHS in Mauritius to guide the starting points in the Double Dichotomous Choice survey questionnaire.

To make sure that the sample is representative of the population, the population was segregated as per the regions, similar to Al-Hanawi et al. [20]. The regions were taken as the 9 different districts over the Island of Mauritius. A random sample of working individuals was chosen from each district, with the sample size being proportional to that of that district. Ultimately, the rural to urban population ratio in Mauritius was respected.

#### 2.12.4. Design of the Questionnaire

The introductory section of the questionnaire lays the main information on the title and purpose of the study to the participants. The questionnaire is divided into four sections. Section A captures data on the respondent’s personal information. Section B captured data about the willingness of contributing annually to the hypothetical scenario.

Section C and section D appraised the planned and perceived behaviour of the respondent towards the contribution. Section E captures information on the respondent’s perception on the different attributes of the public healthcare service based on their rating of satisfaction. Finally, Section D amassed information about the health status of the respondent.

#### 2.12.5. The Hypothetical Scenario

The hypothetical scenario is based on a national health insurance scheme into which income earners are to make a recurrent contribution. The contributions would be used to supplement the government’s healthcare budget to improve the Quality of Public Healthcare Service (QPHS) in Mauritius such as “waiting time before seeing the doctor”, “waiting time before getting appointment with a specialist”, “waiting time for laboratory tests”, “quality of drugs at pharmacy”, “staff attitudes”, “time spent with the doctor to discuss problems and state of health”, etc. It was also made clear that there would be no refund for those who do not use public healthcare services and even those not contributing (non-income earner) would still be benefiting from the improved services.

#### 2.12.6. The Method of Payment

The participants were informed that the payment vehicle is similar to an insurance premium into which citizens are required to make recurrent contributions.

#### 2.12.7. Eliciting Monetary Values

In order to capture WTP, after providing details on the hypothetical scenario and method of payment, the study used the Double-bounded dichotomous choice elicitation question, which is given as follows:

“Would you be willingness to contribute (starting point amount) to the scheme per year to (hypothetical scenario)?

If Yes: And would you pay (higher bid)?

If No: And would you pay (lower bid amount)?”

The double-bounded dichotomous choice elicitation question technique is considered to be superior than “direct open-ended questions” and “bidding games” elicitation techniques as it is relatively more informative about the WTP of the respondents and less costly to be applied [13]. Even compared to single-bounded dichotomous choice, the double-bounded dichotomous choice format is considered to provide more conservative results in experimental analysis [77].

The double-bounded dichotomous choice is based on the presentation of two bids. If the respondent chooses “yes” for the initial bid, then the next bid would be higher; and if the respondent choses “no” for the initial bid, then the next bid would be lower. Similar to other studies [78,79], this study uses different bid combinations using three starting points following: (750/1500/3000), (1500/3000/6000), and (3000/6000/12,000) Mauritian Rupees (MUR). From these combinations, the initial values are the middle values and this corresponds to the initial bids offered to the respondent. Each survey questionnaire had a random starting bid. As such, each respondent was presented a starting bid based on randomness. If the respondent accepted the initial bid, he was presented a higher bid (the amount on the right in the combinations above) and if the respondent declined the initial bid, he was presented a lower bid (the amount on the left in the combinations above) [80,81,82,83].

Originally, a survey was conducted as “a pre-test”, using a samples size of 100 questionnaires using the open-ended format, before conducting the main survey. The result from this “pre-test” survey was used to set the range of bids. In line with other studies, 10% of the values collected during the pre-test were trimmed off on both tails of the bid distribution, after which the three bid combinations were reached in the main survey [81,84].

#### 2.12.8. Altruistic Behaviour

The two elements of Altruistic Behaviour, Subjective Obligation to Pay (SOP) and General Warm Glow (GW), were captured in the questionnaire using 5-point likert scales. In line with Liebe et al. (2011), SOP was measured using the Q30 “You like to contribute money to health projects” and GW was measures using Q31 “You get a good feeling by donating to good causes”. 5-point likert scale with “Strongly Agree”, “Agree”, “Neither Agree Nor Disagree”, “Agree”, “Strongly Agree” were used for both SOP and GW.

#### 2.12.9. Health Risk Attitude

Health Risk Attitude (HRA) was measures using the scale developed by Van Osch and Stiggelbout [29]. The HRA measure consists of six items each followed by a 5-point rating scale ranging from 1 (Strongly disagree) to 5 (Strongly agree) given as follows: Q32: “You think you take good care of your body”; Q33:” It is vital to you that you organize your life so that you can later enjoy good health”; Q34: “If it concerns your health, then you see yourself as someone who avoids risks”; Q35: “Your health means everything to you”; Q36: “Safety first, where your health is concerned” Q37:” To enjoy good health now and in the future, you are prepared to forego a lot”. The average of the items was taken as a direct measure for the relevant variable. The internal consistency for this scale, as evaluated through the Cronbach’s alpha, was 0.878.

#### 2.12.10. Perceived Response Efficacy

Based on previous literature on WTP [35,85,86], the study uses the item Q38 “You have trust that the government can effectively manage the QPHS scheme” to capture Perceived Response Efficacy. Respondents rated on a 5-point scale from 1, representing “strongly disagree” to 5, representing, “strongly agree”.

#### 2.12.11. Perceived Quality

The study measures the status quo perceived quality of the public healthcare services using the 6 items Q39: “Waiting time before seeing a doctor (general)”; Q40: “Waiting time before getting appointment with a specialist”; Q41: “Waiting time for laboratory tests”; Q42: “Quality of drugs at public pharmacy”; Q43: “Attitude of public health staffs” Q44: “Time spent with the doctor to discuss problems and state of health”. Each item was rated on a 5-point scale from 1 (strongly disagree) to 5 (strongly agree). These items have also been captured in Pavel et al. [18] and Al-Hanawi et al. [20]. The average of the items was taken as a direct measure for the relevant variable. The internal consistency for this scale, as evaluated through the Cronbach’s alpha, was 0.848.

#### 2.12.12. Public Goods Theory

Based on the theory of public goods, dilemma concern (DC) and trust in other people’s cooperation (TC) are captured in the model. DC is measured using item Q25: “If others do not take part, you will also not engage in improving QPHS” and TC is measures using item Q26: “You believe others are willing to contribute something to improve QPHS” using the wordings as in Liebe et al. [25]. Respondents rated on a 5-point scale from 1, representing “strongly disagree” to 5, representing, “strongly agree”.

#### 2.12.13. Norm-Activation

Norm Activation is captures using similarly wordings as in Litvine and Wüstenhagen [35]; through Q27: “Compared to other policy measures, improved QPHS is not a high priority”; Q28: “The state is doing enough to improve QPHS; there is no need to contribute”; Q29 “You already contribute enough for other things; you do not have to contribute to improve QPHS” which measures the awareness of need and responsibility for paying. Each item was rated on a 5-point scale from 1 (strongly disagree) to 5 (strongly agree). The mean of the reverse of the respective items were considered to be the direct measure of the corresponding variable. The internal consistency for this scale, as assessed using Cronbach’s alpha, was 0.669.

#### 2.12.14. Planned Behaviour

This study captures the components of the Theory of Planned Behaviour (Attitude, Subjective Norm, Moral Norm and Perceived Behavioural Control) in the same way as López-Mosquera et al. [87]. The average of the items was taken as a direct measure for the relevant variable. Respondents rated on a 5-point scale from 1, representing “strongly disagree” to 5, representing, “strongly agree”.

Attitude was measured by 4, 5-point likert scales: Q13: ‘‘You think the idea of contributing to improve QPHS is beneficial”; Q14: “You think the idea of contributing to improve QPHS is responsible”; Q15: “You think the idea of contributing to improve QPHS is intelligent” and Q16: “You think the idea of contributing to improve QPHS is useful”. Respondents rated on a 5-point scale from 1, representing “strongly disagree” to 5, representing, “strongly agree”. The internal consistency for this scale, as evaluated through the Cronbach’s alpha, was 0.943.

Three statements were used to capture subjective norms: Q17: “People who are important to you think that you should contribute to improve QPHS”; Q18: “People who are important to you would expect you to contribute to improve QPHS”; Q19: “People whose opinions you value would contribute to improve QPHS”. Respondents rated on a 5-point scale from 1, representing “strongly disagree” to 5, representing, “strongly agree”. The internal consistency for this scale, as evaluated through the Cronbach’s alpha, was 0.81.

Moral norm was captured using three statements: Q20: “You feel that you should contribute to improve QPHS”; Q21: “You feel guilty if you don’t contribute to improve QPHS”; Q22: “Contributing to the improvement of QPHS makes you feel like a better person”. Respondents rated on a 5-point scale from 1, representing “strongly disagree” to 5, representing, “strongly agree”. The internal consistency for this scale, as evaluated through the Cronbach’s alpha, was 0.846.

Perceived Behavioural control was evaluated through two statements: Q23: “It would be difficult for you to contribute to improve QPHS”; Q24: “You think that your contribution would not improve QPHS”. Respondents rated on a 5-point scale from 1, representing “strongly disagree” to 5, representing, “strongly agree”. The internal consistency for this scale, as evaluated through the Cronbach’s alpha, was 0.6004.

### 2.13. Contingent Valuation Method and Double Dichotomous Choice Model

The contingent valuation method (CVM) is an approach which considers a survey based on the hypothetical scenario about particular commodities or services and then directly derives the WTP of those commodities or services. The CVM is normally applied to quantify the value of commodities or services, when the latter do not fetch any price similar to public services [88].

In CVM studies, it is common practice and for theoretical validity to estimate an individual’s WTP which can be derived from the income-compensating function [89]. The WTP is the measure of expected benefit and can be influenced by a vector of the individuals’ personal characteristics, experiences about healthcare services and economic conditions. The WTP function of the respondent can be represented as:

WTP(q_1_) = f (P,q_1_, q_0_,Q,M,X′) (7)

where P is the vector of prices for the marketed goods, q_1_ is the healthcare services which are to be improved, q_0_ is the baseline level of the healthcare services of interest, Q is a vector of the other public goods, M is income, and X′ is a vector of the individual’s tastes or personal characteristics. P, Q and q_1_ are assumed to be constant for all individual. Equation (1) provides the foundation to estimate a valuation function that describes the monetary value of a change in economic welfare which arises for any change in q_1_ [90]. Let *i* = 1, …, *N* be the index for each individual. Representing these drivers of WTP as a vector, *x_i_*, and under the assumption of a log-linear functional form, the WTP can be algebraically represented as:

(8) $$\ln\;{\mathrm{WTP}}_{i}^{*}=x_{i}^{\prime }\beta +u_{i}$$

where *x_i_* is a vector of explanatory variables, *β* is a vector of parameters, and $u_{i}\sim N\left(0,\sigma^{2}\right).\;$Based on the hypotheses, the explanatory variables relates to the demographic and socioeconomic characteristics (age, gender, residential area, educational level, income level, family size, civil status and ownership of private health insurance) as well as measures to capture Altruistic Behaviour (General Warm Glow and Subjective Obligation to Pay), Health Risk Attitude, Perceived Response Efficacy, Perceived Quality, Public Goods Theory (Dilemma Concerns and Trust in Others), Norm-Activation and Planned Behaviour (Attitude, Social Norms, Moral Norms, Perceived Behavioural Control).

To conduct the CVM, let *G_i_* be the generic bid for respondent *i* and $G_{i}^{0}$ be the initial bid. If the respondent answers with a “yes” to $G_{i}^{0}$, the upper bound follow-up $G_{i}^{U}>G_{i}^{0}$ is asked, else a lower bound follow-up bid of $G_{i}^{L}<G_{i}^{0}$ is proposed. Indeed, each respondent is faced with two specific bids, and such format results in to four responses: “yes-yes”, “no-no”, “yes-no” and “no-yes”. Let their binary-valued indicator functions are $I_{i}^{YY}$, $I_{i}^{NN}$, $I_{i}^{YN}$, and $I_{i}^{NY}$, respectively, such that:

(9) $$I_{i}^{YY}=I\left(\mathrm{response of respodent i is “yes-yes”}\right)\;I_{i}^{NN}=I\left(\mathrm{response of respodent i is “no-no”}\right)\;I_{i}^{YN}=I\left(\mathrm{response of respodent i is “yes-no”}\right)\;I_{i}^{NY}=I\left(\mathrm{response of respodent i is “no-yes”}\right)$$

where *I*(·) represents an indicator function, which is equivalent to one if the argument is true; else it is zero. Assuming rationality whereby respondents do not agree to pay more than they are willing to do so, then the set of observed bid responses will yield the set of intervals for estimating WTP. Assuming rationality whereby respondents do not agree to pay more than they are willing to do so, then the set of observed bid responses will yield the set of intervals for estimating WTP. The application of maximum likelihood estimation allows the estimation of *β* and *σ* [91]. Let Φ(·) be the standard normal cumulative distribution function, the log-likelihood function that needs to be maximized in order to find the parameters of the Equation (1) is shown as follows:


(10) $$\ln\ell =\overset{N}{\underset{i=1}{\sum }}\left\{\begin{array}{c} I_{i}^{YY}\ln\left[1-\Phi \left(\frac{\beta_{i}^{H}-x_{i}^{\prime }\beta }{\sigma }\right)\right]+I_{i}^{NN}\ln\left[1-\Phi \left(\frac{\beta_{i}^{L}-x_{i}^{\prime }\beta }{\sigma }\right)\right]+\\ I_{i}^{YN}\ln\left[\Phi \left(\frac{\beta_{i}^{H}-x_{i}^{\prime }\beta }{\sigma }\right)-\Phi \left(\frac{\beta_{i}-x_{i}^{\prime }\beta }{\sigma }\right)\right]\\ +I_{i}^{NY}\ln\left[\Phi \left(\frac{\beta_{i}-x_{i}^{\prime }\beta }{\sigma }\right)-\Phi \left(\frac{\beta_{i}^{L}-x_{i}^{\prime }\beta }{\sigma }\right)\right] \end{array}\right\}$$

The WTP can be computed by the *β* and *σ*.

### 2.14. Hierarchical Model

Hierarchical modelling is mainly used to test theoretical hypotheses. This method allows to examine the effect of different independent variables in a sequential way. It captures “hierarchical effects” where explanatory variables are captured at different levels [92]. Different regressions are modelled, whereby additional variables to the preceding model at each successive step, to capture the contribution of the additional set of variables.

The purpose of hierarchical modelling is to capture the incremental importance of each set of variables [93]. The relative importance of each independent variable can be determined by the how much it contributes to the prediction of a criterion [94,95]. This incremental importance is reflected by the improvement in the $R^{2}$ or adjusted $R^{2}$, which measure the proportion of variation in the dependent variable explained by the model, following the inclusion of a set of variables [96,97,98]. Therefore, the main measurement relies on change in $R^{2}$ (∆ adjusted $R^{2}$) or adjusted $R^{2}$ (∆ adjusted $R^{2}$) when applying hierarchical regression [96,99].

The hierarchical model explores the determinants of WTP. Demographic and socioeconomic characteristics were first entered in the model (Model 1) as control variables. Successively, the variable predictors of WTP were entered in the model in the following order, Altruistic Behaviour (in Model 2), Health Risk Attitude (in Model 3); Perceived Response Efficacy (Model 4); Perceived Quality (Model 5); Public Goods Theory (Model 6); Norm-Activation (Model 7); Planned Behaviour (Model 8), as given in Table 3.

## 3. Data Analysis

### 3.1. Quality Attributes of Public Health Care Services

As given in Table 3, the mean value of “Perceived Quality” is 2.568 and varies between 1 to 5. This shows that on average people are more satisfied with the QPHS in Mauritius. This is in line with previous findings of Sobhee [7] and Ramsaran-Fowdar [8].

Table 4 summarizes the responses of respondents for the corresponding questions on the attributes of the QPHS in Mauritius. It is observed that for all the 6 attributes assessed, less than 4% of respondents were “very satisfied” with QPHS, and less than 30% of respondents were at least satisfied with QPHS.

For “Attitude of public health staffs” and “Time spent with the doctor to discuss problems and state of health” less than 50% of respondents were dissatisfied. Most dissatisfaction came from “Waiting time before getting appointment with a specialist” and “Waiting time before seeing doctor” where 61% and 51% were at least dissatisfied. For these two attributes, more than 20% of respondents were “Very Dissatisfied”. Respondents show less dissatisfaction towards “Quality of drugs at public pharmacy”. As such, this flags the attributes of the public healthcare system where policymakers should focus more attention.

### 3.2. Empirical Results

#### 3.2.1. Estimated WTP

The mean annual WTP is estimated to be Rs 1482 (37 USD). In Mauritius, according to official data, average monthly income for employees is estimated to be Rs 22,600 (571 USD) in 2019 [72]. This indicates that WTP accounts for 0.5% of the average annual income of working people in Mauritius. Extending the mean annual WTP value to the working population of Mauritius, Mauritian households are willing to pay Rs 817 million (21million USD) per annum for improving quality of public healthcare services.

#### 3.2.2. Demographic and Socioeconomic Characteristics

Table 5 provides the results for the hierarchical DBDC models. Similar to Jaunky et al. [98], eight models are run and the implications of each hypothesis as depicted in Figure 3 are assessed. For Model 1, the demographic and socioeconomic characteristics explained 3.5% of the variance in WTP.

In all the Models, Age is consistently negative and significant (*p* < 0.05), implying that probability of WTP for the improving QPHS decreases with the increase in age. This result is consistent with other studies [18,20]. No non-linear relationship between age and the dependent variable is uncovered as the variable Age squared is found to be statistically insignificant (*p* = 0.79) at conventional levels.

The coefficient on “Rural” has a positive and significant sign (*p* < 0.01), consistent in all the models, which implies that individuals residing in rural areas are more likely to pay to improve QPHS. While Pavel et al. [18] finds the effect of location to be insignificant, Al-Hanawi et al. [20] finds that the sign of location varies across the quality improvement concerned. Rural inhabitants can be expected to have higher health expenditures, such as out-of-pocket expenditures on prescription medications [100], than urban population. This could be due to the higher occurrence of poor health status in the rural population and to inferior quality to preventive care in rural areas [101]. This could influence a greater need for better quality service for rural inhabitants.

The coefficients of “Undergraduate” and “Post-Graduate” are positive and significant across all the models, with that “Post-Graduate” being higher than that of “Post-Graduate”, except in Model 8 where the coefficient of “Undergraduate” is not statistically significant. This suggests that relative to individuals with secondary level of education, individuals with undergraduate level of education are more likely to pay and individuals with postgraduate level of education are in turn even more likely to pay than those with undergraduate level of education. Similarly, the coefficients of “Income 3” and “Income 4” are positive and significant across all the models (*p* < 0.01), with that “Income 4” being higher than that of “Income 3”. This indicates that compared to individuals with Income 1, individuals with Income 3 are more likely to pay and individuals with Income 4 are in turn even more likely to pay than those with Income 3. Both Pavel et al. [18] and Al-Hanawi et al. [20] also find that individuals with higher education and higher income are more likely to pay to improve QPHS.

Individuals being under and insurance scheme were coded as 1 while those who were not under a medical scheme were coded as 0. Form the results, the coefficient of “Insurance” is positive and significant in all models. This shows that individuals under private medical insurance schemes are more likely to pay to improve QPHS than that those not under medical insurance scheme. Al-Hanawi et al. [20] controlled for medical insurance but the same coefficient was statistically insignificant across all its estimations.

#### 3.2.3. Altruistic Behaviour

Altruistic Behaviour accounted for an additional 0.6% of the variance in WTP. In Model 2, the coefficients of both “General Warm Glow” and “Subjective Obligation to Pay” are as expected positive [25] and significant (*p* < 0.05), while the same coefficients are statistically insignificant in the rest of the models. As such the study finds no robust evidence to show that the warm glow motivation is prevalent in the survey. Further, as per Adamowicz, et al. [102], “Warm glow” exist when respondents claim that they would pay money to improve the status quo, unconditional on the details of the specific hypothetical scenario under study. Indeed, the lack of robustness in the effect of “General Warm Glow” is a plus point as the commonly cited NOAA panel on Contingent Valuation, which is a panel of high profile economists (chaired by Nobel Prize laureates Kenneth Arrow and Robert Solow) which was convened under the auspices of the National Oceanic and Atmospheric Administration (NOAA) in 1993, recommends that “survey should be designed to deflect the general warm-glow of giving or the dislike of big business away from the specific environmental program that is being evaluated” [103].

Based on the above, “Hypothesis 1a: People who perceive a general obligation to support good causes are more willing to pay for improving quality of healthcare services” and “Hypothesis 1b: People who perceive a subjective obligation to pay for health projects are more willing to pay for improving quality of healthcare services” cannot be accepted.

#### 3.2.4. Health Risk Attitude

From Model 3, “Health Risk Attitude” is found to have an incremental value of 0.13% of the variance in WTP. “Health Risk Attitude” is positive and statistically significant (*p* < 0.10) in Model 3 only, while in the rest of the models the coefficients of “Health Risk Attitude” are statistically insignificant.

Therefore, “Hypothesis 2: The risk attitude of people affects their WTP for improving quality of healthcare services” cannot be accepted.

#### 3.2.5. Perceived Response Efficacy (PRE)

Model 4 adds PRE to the estimated model. It contributes for an additional 1.9% of the variance in WTP. As expected, the coefficient of PRE is positive and statistically significant, across all the Models where it is included. This indicates that when individuals perceive the recommended solution as effective, they are more likely to pay to improve QPHS. Similar to this finding, Habibov et al. [19] find that higher “social trust” leads to higher willingness to pay taxes to enhance public healthcare.

Thus, Hypothesis 3: Perceived response efficacy is positively related to the WTP for improving quality of healthcare services is accepted.

#### 3.2.6. Perceived Quality

Perceived Quality contributes to 0.18% of the variance in WTP as shown in Model 5. The coefficient of same is negative and statistically significant in Model 5 to 7. The previous literature [18,20] emphasized on this element and also report an inverse relationship between perceived quality of attributes of healthcare services and WTP. This indicates that when individuals perceived QPHS to be poorer they are more likely to pay to improve QPHS. In the same way, individuals who are more satisfied with the quality of public healthcare services are less likely to contribute to its improvement.

Given the above, “Hypothesis 4: Perceived Quality of Public Healthcare Services is negatively related to the WTP for improving quality of healthcare services” is accepted.

#### 3.2.7. Public Good Theory

Following the inclusion of “Dilemma Concern” and “Trust in other people’s cooperation” increases the model explained an additional of 1% of the variance in WTP. The coefficient of “Dilemma Concern” is statistically significant in all the models (6, 7 and 8) while that of “Trust in other people’s cooperation” is statistically significant in models 6 and 7 only. The sign of the coefficient of both variables are as expected; where this sign of the coefficient of “Dilemma Concern” is negative and that of “Trust in other people’s cooperation” is positive. The negative impact of “Dilemma Concern” can be interpreted as the more people improvement in the healthcare service as a social dilemma, the less likely they are willing to pay to improve QPHS [25].

Given the inconsistency in the statistically significant of the coefficient for “Trust in other people’s cooperation” and the consistency of the coefficient for “Dilemma Concern”, “Hypothesis 5a: The more people improvement in the healthcare service as a social dilemma, the less likely they are willing to pay for its improvement” is accepted but “Hypothesis 5b: People who trust that others will cooperate are more willing to pay for improving quality of healthcare services” cannot be accepted.

#### 3.2.8. Norm Activation

When norm-activation is added to the Model 7, the Pseudo R^2^ increases by 0.0269. In both Model 7 and 8, the coefficient of Norm-Activation is positive and statistically significant (*p* < 0.01). This implies that higher awareness of the need and responsibility for paying increases the likelihood of paying to improve quality of healthcare services are more likely to pay for its improvement is accepted.

It is concluded that Hypothesis 6: People with higher awareness of the need and responsibility for paying to improve quality of healthcare services are more likely to pay for its improvement is accepted.

#### 3.2.9. Theory of Planned Behaviour

When the components of TPB are included in the model, given in Model 8, the Pseudo R^2^ increases by 44.20%. The coefficients of Attitude, Social Norms, Moral Norms and Perceived Behavioural Control are all positive and statistically. It is noted that the coefficient of Attitude, Social Norms and Perceived Behavioural Control are significant at 1% significance level while the coefficient of Moral Norms is statistically significant at 10% significance level. The increased ability of the TPB to predict WTP to improve QPHS reinforces the significance of including measures such as attitude, subjective norms, and perceived behavioural control in WTP experiments.

Attitude is the strongest predictor in the study, having a larger regression coefficient than all other TPB constructs, following by subjective norm, perceived behavioural control and moral norms. This shows that as attitudes, Subjective Norms, Moral Norms and Perceived Behavioural Control of payment for the improvement of quality of public healthcare services become more positive, a person’s WTP increases.

Therefore, the hypotheses: “Hypothesis 7a: As attitudes towards payment for the improvement of quality of public healthcare services become more positive, a person’s WTP increases”; “Hypothesis 7b: As Subjective Norms regarding payment for the improvement of quality of public healthcare services become more positive, a person’s WTP increases”; “Hypothesis 7c: As Moral Norms concerning payment for the improvement of quality of public healthcare services become more positive, a person’s WTP increases”; and “Hypothesis 7d: As Perceived Behavioural Control of payment for the improvement of quality” are all accepted.

## 4. Discussion

The respondents’ mean WTP value was estimated to be Rs 1482 (37 USD) per annum through the double-bounded dichotomous CVM assessment. Among the control variables, age, education, income and being under a medical scheme are shown to affect the WTP value. In particular, the WTP value increases with income and education level, while it decreases with the respondents’ age and being under medical insurance. Further, among the theories applied to explore the determinants of the WTP value, “Perceived Response Efficacy”, “Perceived Quality”, “Social Dilemma” under the Public Good theory, “Norm-Activation” and Theory of Planned Behaviour are found to be important predictors of WTP to improve QPHS. The inclusion of these constructs has improved the predictive power of the theoretical framework in determining the WTP. This might have interesting policy consequences as they allow an accurate insight on WTP.

The integration of the TPB as a Psychometric measure in the estimation provided interesting insights on the behavioural aspects of WTP to improve QPHS. It shows the usefulness and applicability of TPB in determining the WTP. Understanding motives behind values is important for a correct assessment of values themselves. Understanding motives behind values is important for a correct assessment of values themselves. The present research has proved the usefulness and applicability of TPB in determining the WTP to improve QPHS in Mauritius. The constructs of the extended Theory of Planned Behaviour are the strongest predictor of WTP in this study, where the expanded TPB construct explained 4.47% of the variance in the model. This provides support for the efficacy of an extended TPB model, for predicting WTP for improving quality of public healthcare services in Mauritius. TPB indicators were all significant, therefore suggesting that all four components of TPB explain part of WTP to improve QPHS. Within TPB, the least powerful predictors are Moral and Social Norms while attitude and perceived behavioural control are the most powerful predictor. Similar to other studies on WTP, the results obtained show a correct use of the Theory of Planned Behavior (TPB) methodology to explain this conduct [104,105,106,107,108,109,110,111,112,113] These studies have used similar variables and construct to capture TPB, including attitudes, subjective norms and perceived behavioural control This supports the “reliability” of the results and suggests that the Theory of Planned Behaviour is appropriate to model individuals’ WTP as it may enhance the explanatory power of DBDC models. In summary, the current study provides some evidence that the TPB model might represent a useful framework for predicting WTP.

Norm-Activation also predicts respondents’ behaviour quite well, accounting for 2.69% of the of the variance in the model. After the constructs of the Theory of Planned Behaviour, the coefficient for Norm-Activation has a larger regression coefficient than all other constructs at each step of the model. It is noted that similar to this study [25] have also used “awareness of need” and “awareness of responsibility” as variables to capture Norm-Activation.

The results suggest that people with higher awareness of the need and responsibility for paying to improve quality of healthcare services are more likely to pay for its improvement in Mauritius. As such, strategies which emphasise the promotion awareness of the need and responsibility for paying to improve quality of healthcare services will be important in improving QPHS in Mauritius. Furthermore, participants in our study scored high for “Norm-Activation” indicating that the level of awareness for the need and responsibility for paying is already above average.

As expected, perceived response efficacy (PRE) is positively related to the WTP for improving quality of healthcare services in Mauritius. To a lower extent, perceived response efficacy contributes to 1.6% of the variance in WTP. This indicates that when individuals perceive the recommended solution as effective, they are more likely to pay to improve QPHS. Perceived Response Efficacy was captured using the element of trust of the respondent that the government can effectively manage the QPHS scheme. Similar to this finding, Habibov et al. [19] find that an increase in social trust is associated with a greater willingness to pay more taxes to improve public healthcare. From the data gathered the PRE average value is 3.2 implying that on average the respondent perceives this hypothetical scenario scheme as effective in improving quality of public healthcare services in Mauritius. When devising policies in improving QPHS it is important to consider the role of perceived response efficacy when making decisions about taxes for healthcare. If people perceive that a certain policy is not effective in improving the QPHS, they might be less willing to support the initiative. As such, it is recommended that the government should show and reassure that there will be transparency and accountability, in the management of the funding for improving QPHS in Mauritius to build confidence about a proper management and auditing system.

Perceived Quality explains 0.18% of the model. As expected, Perceived Quality of Public Healthcare Services is negatively related to the WTP for improving quality of healthcare services in Mauritius. It is noted that the average value of the PQ index 2.568 which indicates an average level of respondents are slightly more satisfied than neutral over the satisfaction level with the quality of public healthcare services in Mauritius. To improve the QPHS, it is important for policymakers to come up with an indicator to measure quality in the public healthcare system to track evolution of quality and compare with benchmarks similar to the OECD indicator of quality in the healthcare system, with the aim improving the culture of quality and continuous improvement in the public healthcare service.

It is found that most respondents are dissatisfied with the waiting time before seeing a doctor and before acquiring an appointment with a specialist. The implication of this finding is that policies should be directed towards increasing the number of doctors and specialists in the public healthcare sector. The government has already emphasized on the number of doctors working in the Ministry of Health and Wellness increased from 1000 at the end of 2012 to 1568 in 2019. However, though the number of cases seen for treatment in the public healthcare sector exceeded the number in the private healthcare sector by far, less than 50% of doctors were employed in the public healthcare sector.

Out of the 1568 doctors employed by the public healthcare sector, 354 were specialists. 1722 doctors were engaged in private practice, of which 980 were specialists. This represents only 47.7% of doctors employed in the public sector. In 2019, the number of admissions (including re-admissions) to government hospitals in the Island of Mauritius was 194,659 and a total of 5.46 million cases were seen by doctors at the out-patient service points in the public sector. With the higher number of doctors and specialists compared to the public healthcare sector, the total number of cases seen for treatment, including admissions, at the 19 private hospitals and clinics having in-patient services was only 281,056 in 2019.

In addition, from the Theory of Public Good, the result indicates that the more people improvement in the healthcare service as a social dilemma, the less likely they are willing to pay for its improvement. Based on the data gathered, the average value for the “social dilemma” index is 2.634. This shows that on average respondent are quasi-neutral when it comes to assessment the social dilemma concern in improving quality of public healthcare services in Mauritius. The Social dilemma is often regarded as a “collective action problem”. In the literature, motivational solutions can be used to promote in social dilemmas. Policy maker should reinforce the sense of civic responsibility for cooperation for the betterment of public health. The Government of Mauritius has recently come up with the Nine Years of Continuous Basic Education reform which makes provision for all students to successfully complete nine years of basic schooling. The latter incorporates the reinforcing a strong sense of civic responsibility. Civic values should be further extended to the adult population.

## 5. Strength and Limitations

The paper uses the double-bounded dichotomous choice model to model the willingness of Mauritians people to pay to improve the quality of public healthcare services. the model specification has used the hierarchical model to capture “hierarchical effects”; to capture the contribution of the additional set of explanatory variables [92]. As discussed earlier, in contrast to earlier studies [18,20], the study uses different theories: Theory of Planned Behaviour, Schwartz’s Norm-Activation Model and the Theory of Public Goods.

The study uses the discrete choice format in the contingent valuation survey which consists of asking a bid to the respondent with a question, followed by a second bid to the respondent, greater than the first bid if the respondent answers yes to the first bid and lower otherwise. This format of survey is strongly recommended by the work of the NOAA panel [103]. Indeed, one advantage of the discrete choice format is that it mimics the decision-making task that individuals face in everyday life since the respondent accepts or refuses the bid proposed. However, the disadvantage of the double-bounded model is that subject’s responses to the second bid may be influenced by the first bid proposed. This is the so called starting-point bias [114].

## 6. Conclusions

The purpose of the study was to employ a Contingency Valuation (CV) to investigate the willingness of Mauritians people to pay to improve the quality of public healthcare services and the associated determinants using the double-bounded dichotomous choice model. While most studies only take into account a single theory, this study considered competing theories, including Theory of Planned Behaviour, Schwartz’s Norm-Activation Model and the Theory of Public Goods. A drop off survey with a sample size of 974 respondents from the working population is used. The results indicate that the majority of the sample was willing to pay for improving quality of public healthcare services in Mauritius. The results of this study might be of use to policymakers to help with both priority setting and fund allocation.

Other than the conventional determinants of respondents’ demographic and socioeconomic characteristics, the findings support the assertion that psycho-social constructs such as the Theory of Planned Behaviour, Norm-Activation, Public Good Theory, and Perceived Response Efficacy are shown to significantly affect WTP.

## Figures and Tables

**Table 1:** Sustainable Development Goals 2030.

Variable	Global Target	Mauritius in 2015	Mauritius in 2019
Under 5 Mortality Rate	25 per 1000 live births	15.5 per 1000 live births	16 per 1000 live births
Neonatal Mortality Rate	12 per 1000 live births	9.5 per 1000 live births	10.3 per 1000 live births
Maternal Mortality Rate	70 per 100,000 live births	47 per 100,000 live births	62 per 100,000 live births
HIV Incidence Rate	To end by 2030	20.8 per 100,000 mid-year population	28.9 per 100,000 mid-year population
Malaria Incidence Rate	To end by 2030	2.5 per 100,000 mid-year population	3.3 per 100,000 mid-year population
Tuberculosis Incidence Rate	To end by 2030	10.1 per 100,000 population is low	9.0 per 100,000 population is low

Source: Ministry of Health and Quality of Life [4] and Ministry of Health and Wellness [3].

**Table 2:** Summary Empirical Studies on WTP to improve Quality of Healthcare Services.

Author	Sample	Methodology	Major Findings
Pavel et al. [18]	252 patients in Bangladesh	Partial Tobit regression model	Three attributes of a healthcare system have been identified for which higher satisfaction increases patients’ WTP These are: “a closer doctor-patient relationship”; “increased drug availability”; and “increased chances of recovery”. Among these attributes, patients found “the doctor patient relationship” to be the most important and same have the highest WTP.
Habibov et al. [19]	29,526 individuals from 29 post-communist countries	Binomial Probit regression and Instrumental Variables Probit regression models	Higher “Social Trust” leads to higher WTP for more taxes to enhance public healthcare.
Al-Hanawi et al. [20]	1187 heads of household in Saudi Arabia	Partial Tobit regression Model	Respondents’ demographic and socioeconomic characteristics, and “quality attributes of public health care services”, affect WTP for quality improvement.

Source: Authors’ Own Computation.

**Table 3:** Summary Statistics.

Variables	Mean	Standard Deviation	Minimum	Maximum
Dependent:				
Bid1	3482.033	1870.124	1500	6000
Bid2	3129.363	2490.491	750	12,000
Independent:				
Age	38.350	12.044	18	65
Gender:				1
Female (R)	0.553	0.497	0	1
Male	0.447	0.497	0	
Residential Area:				1
Urban (R)	0.431	0.496	0	1
Rural	0.569	0.496	0	
Educational Level:				1
Secondary (R)	0.243	0.429	0	1
Diploma	0.176	0.381	0	1
Urban	0.393	0.489	0	1
Post-Graduate	0.188	0.391	0	
Income Level:				1
Income1: Rs.10200-Rs.20000 (R)	0.332	0.471	0	1
Income2: Rs.20001-Rs.30000	0.265	0.441	0	1
Income3: Rs.30000-Rs.40000	0.194	0.396	0	1
Income4: Above Rs.40000	0.209	0.407	0	15
Family Size	3.867	1.338	1	
Civil Status:				1
Single (R)	0.294	0.456	0	1
Married	0.674	0.469	0	1
Divorce	0.021	0.142	0	1
Others	0.005	0.072	0	1
Medical Insurance	0.249	0.433	0	
Altruistic Behaviour:				5
General Warm Glow	3.760	0.899	1	5
Subjective Obligation to Pay	3.321	1.027	1	5
Health Risk Attitude	3.910	0.704	1	5
Perceived Response Efficacy	3.213	0.855	1	5
Perceived Quality	2.568	0.806	1	
Public Goods Theory:				5
Dilemma Concerns	2.634	1.133	1	5
Trust in Others	3.034	0.918	1	5
Norm-Activation	3.244	0.860	1	
Planned Behaviour:				5
Attitude	3.374	1.059	1	5
Social Norms	3.089	0.933	1	5
Moral Norms	2.995	0.972	1	5
Perceived Behavioural Control	3.091	0.931	1	

Note: R denotes reference category.

**Table 4:** Summary of responses on attributes.

Corresponding Question	Attribute	Very Dissatisfied	Dissatisfied	Neutral	Satisfied	Very Satisfied
Q39	Waiting time before seeing doctor	20.74%	30.49%	31.11%	16.22%	1.44%
Q40	Waiting time before getting appointment with a specialist	29.47%	31.93%	23.92%	12.32%	2.36%
Q41	Waiting time for laboratory tests	19.71%	30.90%	29.16%	17.86%	2.36%
Q42	Quality of drugs at public pharmacy	15.09%	21.15%	34.19%	26.80%	2.77%
Q43	Attitude of public health staffs	16.63%	23.51%	36.14%	21.56%	2.16%
Q44	Time spent with the doctor to discuss problems and state of health	16.94%	28.75%	29.98%	20.94%	3.39%

**Table 5:** Double Bounded Dichotomous Choice Hierarchical Modelling.

Variables	(1)	(2)	(3)	(4)	(5)	(6)	(7)	(8)
Age	−0.009	−0.010	−0.011	−0.011	−0.010	−0.011	−0.009	−0.010
	(0.004) **	(0.004) **	(0.004) **	(0.004) **	(0.004) **	(0.004) **	(0.004) **	(0.004) **
Gender:								
Male	0.037	0.056	0.060	0.026	0.041	0.026	0.035	0.018
	(0.077)	(0.077)	(0.077)	(0.076)	(0.076)	(0.075)	(0.074)	(0.071)
Residential Area:								
Rural	0.312	0.318	0.313	0.296	0.296	0.283	0.273	0.262
	(0.077) ***	(0.077) ***	(0.077) ***	(0.075) ***	(0.076) ***	(0.075) ***	(0.074) ***	(0.071) ***
Educational Level:								
Diploma	0.064	0.066	0.068	0.067	0.073	0.073	0.063	−0.029
	(0.122)	(0.122)	(0.122)	(0.120)	(0.120)	(0.118)	(0.117)	(0.113)
Undergraduate	0.202	0.182	0.184	0.186	0.182	0.176	0.168	0.126
	(0.105) *	(0.105) *	(0.105) *	(0.103) *	(0.103) *	(0.102) *	(0.100) *	(0.096)
Post-Graduate	0.310	0.278	0.283	0.296	0.302	0.338	0.295	0.239
	(0.125) **	(0.125) **	(0.125) **	(0.123) **	(0.123) **	(0.122) **	(0.120) **	(0.116) **
Income Level:								
Income2: Rs.20001-Rs.30000	0.146	0.151	0.152	0.123	0.119	0.128	0.104	0.135
	(0.105)	(0.105)	(0.105)	(0.103)	(0.103)	(0.102)	(0.100)	(0.096)
Income3: Rs.30000-Rs.40000	0.469	0.451	0.449	0.401	0.399	0.422	0.418	0.436
	(0.124) ***	(0.123) ***	(0.123) ***	(0.121) ***	(0.122) ***	(0.120) ***	(0.118) ***	(0.114) ***
Income4: Above Rs.40000	0.613	0.574	0.575	0.530	0.531	0.548	0.488	0.552
	(0.137) ***	(0.137) ***	(0.137) ***	(0.135) ***	(0.135) ***	(0.134) ***	(0.131) ***	(0.127) ***
Family Size	−0.033	−0.037	−0.037	−0.039	−0.037	−0.038	−0.040	−0.036
	(0.028)	(0.028)	(0.028)	(0.027)	(0.027)	(0.027)	(0.026)	(0.025)
Civil Status:								
Married	0.064	0.053	0.059	0.068	0.068	0.056	0.059	0.054
	(0.100)	(0.100)	(0.100)	(0.098)	(0.098)	(0.097)	(0.096)	(0.092)
Divorce	−0.005	−0.083	−0.093	−0.104	−0.085	−0.119	−0.168	−0.190
	(0.286)	(0.288)	(0.288)	(0.283)	(0.283)	(0.281)	(0.275)	(0.263)
Others	−0.590	−0.593	−0.551	−0.277	−0.239	−0.107	−0.056	−0.137
	(0.534)	(0.532)	(0.531)	(0.519)	(0.521)	(0.517)	(0.507)	(0.513)
Insurance	0.167	0.176	0.167	0.166	0.158	0.156	0.175	0.176
	(0.087) *	(0.087) **	(0.087) *	(0.086) *	(0.086) *	(0.085) *	(0.083) **	(0.080) **
Altruistic Behaviour:								
General Warm Glow	-	0.105	0.073	−0.003	−0.006	−0.014	−0.029	−0.060
	-	(0.047) **	(0.051)	(0.052)	(0.052)	(0.051)	(0.051)	(0.050)
Subjective Obligation to Pay	-	0.073	0.065	−0.001	0.004	0.016	0.032	0.036
	-	(0.041) **	(0.041)	(0.042)	(0.042)	(0.041)	(0.041)	(0.040)
Health Risk Attitude	-	-	0.101	0.043	0.043	0.031	0.026	−0.066
	-	-	(0.061) *	(0.061)	(0.061)	(0.061)	(0.060)	(0.059)
Perceived Response Efficacy	-	-	-	0.323	0.334	0.278	0.227	0.095
	-	-	-	(0.051) ***	(0.052) ***	(0.054) ***	(0.053) ***	(0.053) *
Perceived Quality	-	-	-	-	−0.091	−0.097	−0.062	−0.083
	-	-	-	-	(0.047) *	(0.046) **	(0.046)	(0.044) *
Public Goods Theory:								
Dilemma Concerns	-	-	-	-	-	−0.135	−0.075	−0.061
	-	-	-	-	-	(0.033) ***	(0.033) ***	(0.033) *
Trust in Others	-	-	-	-	-	0.101	0.095	−0.067
	-	-	-	-	-	(0.044) **	(0.043) **	(0.045)
Norm-Activation	-	-	-	-	-	-	0.341	0.165
	-	-	-	-	-	-	(0.046) ***	(0.048) ***
Planned Behaviour:								
Attitude	-	-	-	-	-	-	-	0.223
	-	-	-	-	-	-	-	(0.053) ***
Social Norms	-	-	-	-	-	-	-	0.190
	-	-	-	-	-	-	-	(0.058) ***
Moral Norms	-	-	-	-	-	-	-	0.096
	-	-	-	-	-	-	-	(0.056) *
Perceived Behavioural Control	-	-	-	-	-	-	-	0.126
	-	-	-	-	-	-	-	(0.043) ***
Constant	7.171	6.533	6.305	6.030	6.197	6.508	5.297	5.276
	(0.213) ***	(0.270) ***	(0.305) ***	(0.304) ***	(0.315) ***	(0.334) ***	(0.373) ***	(0.370) ***
Log-Likelihood	−1038.598	−1032.144	−1030.764	−1010.292	−1008.364	−997.441	−968.471	−920.539
Observations	974	974	974	974	974	974	947	974
Wald χ^2^	74.88	85.93	88.27	124.20	127.03	146.22	191.87	261.23
Prob χ^2^	0.000 ***	0.000 ***	0.000 ***	0.000 ***	0.000 ***	0.000 ***	0.000 ***	0.000 ***
R^2^	0.0356	0.0416	0.0429	0.0619	0.0637	0.0738	0.1007	0.1452
∆R^2^	-	0.0060	0.0013	0.0190	0.0018	0.0101	0.0269	0.0445

Note that ***, ** and * denote 1%, 5% and 10% levels of significance, respectively. The standard errors are in brackets. The R^2^ is the McFadden’s Pseudo R^2^.

## Data Availability

The data presented in this study are available on request from the corresponding author. The data are not publicly available due to confidentiality and data protection issues involved.

## References

[1] Ministry of Health and Quality of Life (2017). National Health Accounts 2017.

[2] World Health Organization World Health Report, 2000: Health Systems: Improving the Performance. https://www.who.int/whr/2000/en/whr00_en.pdf.

[3] Ministry of Health and Wellness Health Sector Strategic Plan. https://health.govmu.org/Communique/HSSP%20Final%2015%20September%202020.pdf.

[4] Ministry of Health and Quality of Life (2016). Health Statistics Report 2015.

[5] World Bank (2020). World Development Indicators 2020.

[6] Walshe K., Smith J. (2011). Healthcare Management.

[7] Sobhee S.K. (2007). Analysing and evaluating the taxpayer’s demand for merit goods: The case of public sector education and health in Mauritius. Dev. S. Afr..

[8] Ramsaran-Fowdar R.R. (2008). The relative importance of service dimensions in a healthcare setting. Int. J. Health Care Qual. Assur..

[9] Mitchell R.C., Carson R.T., Carson R.T. (1989). Using Surveys to Value Public Goods: The Contingent Valuation Method.

[10] Portney P.R. (1994). The contingent valuation debate: Why economists should care. J. Econ. Perspect..

[11] Diener A., O’Brien B., Gafni A. (1998). Health care contingent valuation studies: A review and classification of the literature. Health Econ..

[12] Klose T. (1999). The contingent valuation method in health care. Health Policy.

[13] Bateman I.J., Carson R.T., Day B., Hanemann M., Hanley N., Hett T., Jones-Lee M., Loomes G., Mourato S., Pearce D.W. (2002). Economic valuation with stated preference techniques: A manual. Economic Valuation with Stated Preference Techniques: A Manual.

[14] Samuelson P.A. (1954). The pure theory of public expenditure. Rev. Econ. Stat..

[15] Schwartz S.H. (1977). Normative influences on altruism. Adv. Exp. Soc. Psychol..

[16] Fishbein M., Ajzen I. (1977). Belief, Attitude, Intention, and Behavior: An Introduction to Theory and Research.

[17] Del Saz-Salazar S., Rausell-Köster P. (2008). A double-hurdle model of urban green areas valuation: Dealing with zero responses. Landsc. Urban Plan..

[18] Pavel M.S., Chakrabarty S., Gow J. (2015). Assessing willingness to pay for health care quality improvements. BMC Health Serv. Res..

[19] Habibov N., Cheung A., Auchynnikava A. (2017). Does social trust increase willingness to pay taxes to improve public healthcare? Cross-sectional cross-country instrumental variable analysis. Soc. Sci. Med..

[20] Al-Hanawi M.K., Vaidya K., Alsharqi O., Onwujekwe O. (2018). Investigating the willingness to pay for a contributory National Health Insurance Scheme in Saudi Arabia: A cross-sectional stated preference approach. Appl. Health Econ. Health Policy.

[21] Jaunky V.C., Jeetoo J., Thomas J.M. (2021). Willingness to pay for the conservation of the Mauritian flying fox. Glob. Ecol. Conserv..

[22] Kahneman D., Knetsch J.L. (1992). Valuing public goods: The purchase of moral satisfaction. J. Environ. Econ. Manag..

[23] Kahneman D., Ritov I., Jacowitz K.E., Grant P. (1993). Stated willingness to pay for public goods: A psychological perspective. Psychol. Sci..

[24] Guagnano G.A., Dietz T., Stern P.C. (1994). Willingness to pay for public goods: A test of the contribution model. Psychol. Science.

[25] Liebe U. (2007). Zahlungsbereitschaft für kollektive Umweltgüter.

[26] Margolis H. (1982). Selfishness Altruism and Rationality: A Theory of Social Choice.

[27] Andreoni J. (1990). Impure altruism and donations to public goods: A theory of warm-glow giving. Econ. J..

[28] Huls S.P., van Osch S.M., Brouwer W.B., van Exel J., Stiggelbout A.M. (2020). Psychometric evaluation of the Health-Risk Attitude Scale (HRAS-13): Assessing the reliability, dimensionality and validity in the general population and a patient population. Psychol. Health.

[29] Van Osch S.M.C. (2007). The development of the health-risk attitude scale. The Construction of Health State Utilities. Ph.D. Thesis.

[30] Himmler S., van Exel J., Perry-Duxbury M., Brouwer W. (2020). Willingness to pay for an early warning system for infectious diseases. Eur. J. Health Econ..

[31] Witte K. (1994). Fear control and danger control: A test of the extended parallel process model. Commun. Monogr..

[32] Maloney E.K., Lapinski M.K., Witte K. (2011). Fear appeals and persuasion: A review and update of the extended parallel process model. Soc. Personal. Psychol. Compass.

[33] Rogers R.W., Cacioppo J., Petty R. (1983). Cognitive and physiological processes in fear appeals and attitude change: A revised theory of protection motivation. Social Psychophysiology.

[34] Eppright D.R., Tanner J.F., Hunt J.B. (1994). Knowledge and the ordered protection motivation model: Tools for preventing AIDS. J. Bus. Res..

[35] Litvine D., Wüstenhagen R. (2011). Helping “light green” consumers walk the talk: Results of a behavioural intervention survey in the Swiss electricity market. Ecol. Econ..

[36] Mostafa A., El Houssinie M., Hussein R.S. (2021). Perceived efficacy of existing waterpipe tobacco warning labels versus novel enhanced generic and waterpipe-specific sets. PLoS ONE.

[37] Whitehead J.C. (1995). Willingness to Pay for Quality Changes: Comparative Statics and Theoretical Interpretations of Empirical Results. Land Econ..

[38] Hynes S., Campbell D., Howley P. (2011). A holistic vs. an attribute-based approach to agri-environmental policy valuation: Do welfare estimates differ?. J. Agric. Econ..

[39] Whitehead J.C. (2006). Improving willingness to pay estimates for quality improvements through joint estimation with quality perceptions. South. Econ. J..

[40] Johnson R.E., Rosen C.C., Djurdjevic E., Taing M.U. (2012). Recommendations for improving the construct clarity of higher-order multidimensional constructs. Hum. Resour. Manag. Rev..

[41] Ophuis P.A.O., Van Trijp H.C. (1995). Perceived quality: A market driven and consumer oriented approach. Food Qual. Prefer..

[42] Nikhashemi S.R., Valaei N., Tarofder A.K. (2017). Does brand personality and perceived product quality play a major role in mobile phone consumers’ switching behaviour?. Glob. Bus. Rev..

[43] Olson M. (1965). The Logic of Collective Action: Public Goods and the Theory of Groups.

[44] Sandler T. (1992). Collective Action. Theory and Applications.

[45] Kollock P. (1998). Social dilemmas: The anatomy of cooperation. Annu. Rev. Sociol..

[46] Udéhn L. (1993). Twenty-five years with the logic of collective action. Acta Sociol..

[47] Ledyard J.O., Kagel J., Roth A. (1995). Public goods: A survey of experimental research. Handbook of Experimental Economics.

[48] Camerer C.F. (2003). Behavioral Game Theory. Experiments in Strategic Interactions.

[49] Ostrom E. (2000). Collective action and the evolution of social norms. J. Econ. Perspect..

[50] Franzen A. (1995). Free-Riding or Contributing? Considerations on the Relationship between Environmental Concern and Behavior. Cooperative Environmental Behavior. Models, Experiences, Measures.

[51] Blamey R. (1998). Contingent valuation and the activation of environmental norms. Ecol. Econ..

[52] Blamey R. (1998). The activation of environmental norms: Extending Schwartz’s model. Environ. Behav..

[53] Sugden R., Bateman I.J., Willis K.G. (1999). Public goods and contingent valuation. Valuing Environmental Preferences.

[54] Schwartz S.H., Howard J.A., Derlega V., Grzelak J. (1982). Helping and cooperation: A self-based motivational model. Cooperation and Helping Behavior.

[55] De Groot J.I., Steg L. (2009). Morality and prosocial behavior: The role of awareness, responsibility, and norms in the norm activation model. J. Soc. Psychol..

[56] Qiao G., Gao J. (2017). Chinese tourists’ perceptions of climate change and mitigation behavior: An application of norm activation theory. Sustainability.

[57] Schultz P.W., Gouveia V.V., Cameron L.D., Tankha G., Schmuck P., Franěk M. (2005). Values and their relationship to environmental concern and conservation behavior. J. Cross-Cult. Psychol..

[58] Guagnano G.A. (2001). Altruism and market-like behavior: An analysis of willingness to pay for recycled paper products. Popul. Environ..

[59] Agag G., Brown A., Hassanein A., Shaalan A. (2020). Decoding travellers’ willingness to pay more for green travel products: Closing the intention–behaviour gap. J. Sustain. Tour..

[60] Ajzen I., Fishbein M. (1977). Attitude-behavior relations: A theoretical analysis and review of empirical research. Psychol. Bull..

[61] Ajzen I. (1991). The theory of planned behavior. Organ. Behav. Hum. Decis. Process..

[62] Rekola E.P.M. (2001). The theory of planned behavior in predicting willingness to pay for abatement of forest regeneration. Soc. Nat. Resour..

[63] Wu T. (2012). Explaining consumers’ willingness to pay for local and organic food using extended theory of planned behavior model. Ph.D. Thesis.

[64] Müller J., Acevedo-Duque Á., Müller S., Kalia P., Mehmood K. (2021). Predictive sustainability model based on the theory of planned behavior incorporating ecological conscience and moral obligation. Sustainability.

[65] Jackson-Smith D., Flint C.G., Dolan M., Trentelman C.K., Holyoak G., Thomas B., Ma G. (2016). Effectiveness of the drop-off/pick-up survey methodology in different neighborhood types. J. Rural. Soc. Sci..

[66] Bernardi R.A., Guptill S.T. (2008). Social desirability response bias, gender, and factors influencing organizational commitment: An international study. J. Bus. Ethics.

[67] Frey B.B. The SAGE Encyclopedia of Educational Research, Measurement, and Evaluation.

[68] Lavrakas P.J. (2008). Social desirability. Encycl. Survey Res. Methods.

[69] Glover D. (2008). Willingness to Pay for the Conservation of Endangered Species in Four Asian Countries.

[70] Ethier R.G., Poe G.L., Schulze W.D., Clark J. (2000). A comparison of hypothetical phone and mail contingent valuation responses for green-pricing electricity programs. Land Econ..

[71] Ma H., Liu H., Gong Y., Jin J., Mao X. (2015). A comparison of mode effects between face-to-face and drop-off contingent valuation surveys. China Agric. Econ. Rev..

[72] Statistics Mauritius Labour Force, Employment and Unemployment–Year 2019. https://statsmauritius.govmu.org/Documents/Statistics/ESI/2020/EI1515/LF_Emp_Unemp_Yr19.pdf.

[73] Raosoft (2014). Sample Size Calculator. http://www.raosoft.com/samplesize.html.

[74] Cooper J.C. (1993). Optimal bid selection for dichotomous choice contingent valuation surveys. J. Environ. Econ. Manag..

[75] Kanninen B.J. (1995). Bias in discrete response contingent valuation. J. Environ. Econ. Manag..

[76] Tyrväinen L., Väänänen H. (1998). The economic value of urban forest amenities: An application of the contingent valuation method. Landsc. Urban Plan..

[77] Soon J.J., Ahmad S.A. (2015). Willingly or grudgingly? A meta-analysis on the willingness-to-pay for renewable energy use. Renew. Sustain. Energy Rev..

[78] Sarkhel P., Banerjee S., Banerjee S. (2016). Willingness to pay before and after program implementation: The case of Municipal Solid Waste Management in Bally Municipality, India. Environ. Dev. Sustain..

[79] Ghosh R., Goyal Y., Rommel J., Sagebiel J. (2017). Are small firms willing to pay for improved power supply? Evidence from a contingent valuation study in India. Energy Policy.

[80] Suzuki T., Abe T., Tsuji S., Shimoda T., Yoshimura S., Ogasawara K. (2019). Survey on the willingness to pay for tele-health consultation. Health Policy Technol..

[81] Zhang L., Fukuda H., Liu Z. (2019). Households’ willingness to pay for green roof for mitigating heat island effects in Beijing (China). Build. Environ..

[82] Xie B.C., Zhao W., Yin Z.L., Xie P. (2019). How much will the residents pay for clean energy? Empirical study using the double bound dichotomous choice method for Tianjin, China. J. Clean. Prod..

[83] Zhu L., Song Q., Sheng N., Zhou X. (2019). Exploring the determinants of consumers’ WTB and WTP for electric motorcycles using CVM method in Macau. Energy Policy.

[84] Cooper P., Poe G.L., Bateman I. (2004). The structure of motivation for contingent values: A case study of lake water quality improvement. Ecol. Econ..

[85] Ajzen I. (2002). Perceived behavioral control, self-efficacy, locus of control, and the theory of planned behavior 1. J. Appl. Soc. Psychol..

[86] Kaiser F.G. (2006). A moral extension of the theory of planned behavior: Norms and anticipated feelings of regret in conservationism. Personal. Individ. Differ..

[87] López-Mosquera N., García T., Barrena R. (2014). An extension of the Theory of Planned Behavior to predict willingness to pay for the conservation of an urban park. J. Environ. Manag..

[88] Venkatachalam L. (2004). The contingent valuation method: A review. Environ. Impact Assess. Rev..

[89] Willig R.D. (1976). Consumer’s surplus without apology. Am. Econ. Rev..

[90] Yoo S.H., Yang H.J. (2001). Application of sample selection model to double-bounded dichotomous choice contingent valuation studies. Environ. Resour. Econ..

[91] Lopez-Feldman A. (2012). Introduction to contingent valuation using Stata.

[92] Garson G.D. (2013). Fundamentals of Hierarchical Linear and Multilevel Modeling. Hierarchical Linear Modeling: Guide and Applications.

[93] Darlington R.B. (1968). Multiple regression in psychological research and practice. Psychol. Bull..

[94] Aron A., Aron E.N. (1999). Statistics for Psychology.

[95] Cohen B.H. (2008). Explaining Psychological Statistics.

[96] Petrocelli J.V. (2003). Hierarchical multiple regression in counseling research: Common problems and possible remedies. Meas. Eval. Couns. Dev..

[97] Johnson R.L., Kellaris J.J., Houston M.J. (1988). An exploratory study of price/perceived-quality relationships among consumer services. NA-Advances in Consumer Research.

[98] Jaunky V.C., Jeetoo J., Bajah C., Ramesh V. (2020). The Importance of Understanding the Anti-Corruption Legislation to Promote Corruption Reporting: Lessons from Mauritius. Int. J. Public Adm..

[99] Wampold B.E., Freund R.D. (1987). Use of multiple regression in counseling psychology research: A flexible data-analytic strategy. J. Couns. Psychol..

[100] Ziller E.C., Coburn A.F., Yousefian A.E. (2006). Out-of-pocket health spending and the rural underinsured. Health Aff..

[101] Lee W.C., Jiang L., Phillips C.D., Ohsfeldt R.L. (2014). Rural-Urban differences in health care expenditures: Empirical data from US households. Adv. Public Health.

[102] Adamowicz W.L. (2004). What’s it worth? An examination of historical trends and future directions in environmental valuation. Aust. J. Agric. Resour. Econ..

[103] Arrow K., Solow R., Portney P.R., Leamer E.E., Radner R., Schuman H. (1993). Report of the NOAA panel on contingent valuation. Fed. Regist..

[104] Shin Y.H., Im J., Jung S.E., Severt K. (2018). Locally sourced restaurant: Consumers willingness to pay. J. Foodserv. Bus. Res..

[105] Yadav R., Pathak G.S. (2017). Determinants of consumers’ green purchase behavior in a developing nation: Applying and extending the theory of planned behavior. Ecol. Econ..

[106] Sánchez M., López-Mosquera N., Lera-López F., Faulin J. (2018). An extended planned behavior model to explain the willingness to pay to reduce noise pollution in road transportation. J. Clean. Prod..

[107] Li Q., Long R., Chen H. (2018). Differences and influencing factors for Chinese urban resident willingness to pay for green housings: Evidence from five first-tier cities in China. Appl. Energy.

[108] Zhang L., Chen L., Wu Z., Xue H., Dong W. (2018). Key factors affecting informed consumers’ willingness to pay for green housing: A case study of Jinan, China. Sustainability.

[109] Grilli G., Notaro S. (2019). Exploring the influence of an extended theory of planned behaviour on preferences and willingness to pay for participatory natural resources management. J. Environ. Manag..

[110] Shan J., Li J., Xu Z. (2019). Estimating ecological damage caused by green tides in the Yellow Sea: A choice experiment approach incorporating extended theory of planned behavior. Ocean. Coast. Manag..

[111] Zahedi S., Batista-Foguet J.M., van Wunnik L. (2019). Exploring the public’s willingness to reduce air pollution and greenhouse gas emissions from private road transport in Catalonia. Sci. Total. Environ..

[112] Judge M., Warren-Myers G., Paladino A. (2019). Using the theory of planned behaviour to predict intentions to purchase sustainable housing. J. Clean. Prod..

[113] Wang Z., Dong X., Yin J. (2018). Antecedents of urban residents’ separate collection intentions for household solid waste and their willingness to pay: Evidence from China. J. Clean. Prod..

[114] Flachaire E., Hollard G. (2006). Controlling starting-point bias in double-bounded contingent valuation surveys. Land Econ..

